# A Novel Approach for Measuring the Burden of Uncomplicated *Plasmodium falciparum* Malaria: Application to Data from Zambia

**DOI:** 10.1371/journal.pone.0057297

**Published:** 2013-02-28

**Authors:** Valerie Crowell, Joshua O. Yukich, Olivier J. T. Briët, Amanda Ross, Thomas A. Smith

**Affiliations:** 1 Department of Epidemiology and Public Health, Swiss Tropical and Public Health Institute, Basel, Switzerland; 2 University of Basel, Basel, Switzerland; 3 Department of Global Health Systems and Development, Tulane University School of Public Health and Tropical Medicine, New Orleans, Louisiana, United States of America; Kenya Medical Research Institute - Wellcome Trust Research Programme, Kenya

## Abstract

Measurement of malaria burden is fraught with complexity, due to the natural history of the disease, delays in seeking treatment or failure of case management. Attempts to establish an appropriate case definition for a malaria episode has often resulted in ambiguities and challenges because of poor information about treatment seeking, patterns of infection, recurrence of fever and asymptomatic infection. While the primary reason for treating malaria is to reduce disease burden, the effects of treatment are generally ignored in estimates of the burden of malaria morbidity, which are usually presented in terms of numbers of clinical cases or episodes, with the main data sources being reports from health facilities and parasite prevalence surveys. The use of burden estimates that do not consider effects of treatment, leads to under-estimation of the impact of improvements in case management. Official estimates of burden very likely massively underestimate the impact of the roll-out of ACT as first-line therapy across Africa. This paper proposes a novel approach for estimating burden of disease based on the point prevalence of malaria attributable disease, or equivalently, the days with malaria fever in unit time. The technique makes use of data available from standard community surveys, analyses of fever patterns in malaria therapy patients, and data on recall bias. Application of this approach to data from Zambia for 2009–2010 gave an estimate of 2.6 (95% credible interval: 1.5–3.7) malaria attributable fever days per child-year. The estimates of recall bias, and of the numbers of days with illness contributing to single illness recalls, could be applied more generally. To obtain valid estimates of the overall malaria burden using these methods, there remains a need for surveys to include the whole range of ages of hosts in the population and for data on seasonality patterns in confirmed case series.

## Introduction

Malaria continues to be a major cause of disability and death in countries where it is endemic [1]. Accurately estimating the burden of morbidity due to the disease is critical for guiding programmatic strategies and resource allocation, and evaluating the impact of malaria control measures. However, commonly-used approaches for estimating malaria burden are problematic as a result of imprecise terminology and estimation techniques that do not allow for the complexity of the natural history of the disease.

**Figure 1:**
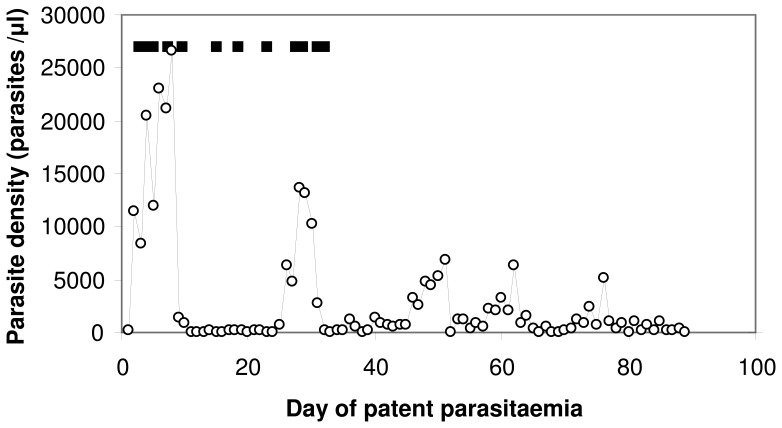
Pattern of parasitaemia and febrile illness in a malaria therapy patient (Patient S-519). ○: Parasite density; ▪ day with fever (core temperature > = 103°F).

Different issues arise in estimating how much morbidity is due to malaria from estimating the mortality burden (which accounts for most of the burden measured in terms of disability-adjusted life years (DALYs) [2]). This paper considers only morbidity. When promptly and effectively treated, malaria illness is of short duration, but if untreated, a single *Plasmodium falciparum* malaria infection can last for many months, causing recurring clinical attacks interspersed with asymptomatic periods [3] during which parasitaemia is often sub-patent. This can be clearly seen in the time courses of parasitaemia and fever observed among neurosyphilis patients treated with malaria therapy. In these studies, the full histories of many patients with untreated malaria infections were recorded following artificial inoculations of malaria parasites given for the purpose of clearing late stage syphilis infections [4]. Figure 1 shows the time pattern of parasitaemia and fever in a neurosyphilis patient treated with *P. falciparum*. In this figure, the single (untreated) infection gives rise to five periods of high parasitaemia. The first two of these are each associated with several bouts of fever indicated by the black bars at the top (see definitions in Table 1).

**Table 1:** Definitions used in this paper.

Term	Definition
Malaria infection	Those parasites descended from a single inoculation of sporozoites#
Incidence of infection	The number of new infection events in a population in unit time
Bout of illness	An uninterrupted set of days during which a patient is considered, or considers himself or herself to be ill for at least part of each day
Malaria episode	A set of bouts of malaria illness considered by the patient or care giver to be of common malaria aetiology*
Incidence of clinical malaria	The rate of new malaria episodes in a population
Point prevalence of clinical malaria	The proportion of the population suffering from symptoms caused by malaria at any one time
Period prevalence of clinical malaria	The proportion of the population suffering from symptoms caused by malaria at any time during a defined interval.
Malaria burden	The morbidity or disability associated with malaria (days with illness, DALYs, or QALYs)

* This definition is intended to capture the way in which the word episode is used, whereby intermittent fever bouts, within a period of continual high parasitaemia characteristically lasting a few weeks (Figure 1), are likely to be considered to be connected. This introduces ambiguities because patients, care givers, and data analysts may disagree about which events are part of the same episode.

# It is debatable whether co-inoculated but genetically distinct parasites should be considered part of the same infection.

This sporadic pattern of clinical symptoms of untreated disease complicates the definition of clinical incidence. For many infectious diseases, for instance influenza, each incident infection leads to one and only one period of illness, or episode. For such diseases, the burden can thus be estimated from the incidence of disease and the duration of episodes, with an appropriate weighting used to convert numbers of episodes into disability adjusted life years (DALYs) or quality adjusted life years (QALYs). By contrast, with malaria, one incident infection may lead to multiple periods of illness (or may be asymptomatic throughout, though it has been claimed that this is unusual [5]). Malaria burden is often expressed in terms of the number of episodes, but it is not clear whether one episode is intended to refer to (i) all illness resulting from a single infection event; (ii) one uninterrupted period of illness; or (iii) all malaria illness within a given period. Infections that are treated promptly and effectively when they first lead to symptoms unambiguously contribute one episode to this total, but when treatment is delayed, or if the infection remains incompletely untreated, it is unclear how many episodes can result from a single infection event. This matters because the disability caused by the disease (and the risk of life-threatening complications) are clearly less when it is treated promptly, but these benefits may be invisible, for instance if incident cases or episodes are counted irrespective of their duration. The term episode clearly refers to some set of bouts, but just how many and which bouts make up an episode is not clear. Table 1 proposes a definition that surmounts this ambiguity.

Statements about incidence of malaria disease are consequently often vague or misunderstood. For instance, the World Health Organization (WHO) estimate of 225 million cases in 2009 [6] is intended to refer to the total number of clinical episodes. The ambiguity in what is meant by an episode makes interpretation difficult. In many countries, mostly outside Africa, burden is reported using passive case detection data, and in WHO statistics, estimates of morbidity rates for these countries are corrected for reporting completeness, diagnostic error, and attendance rates [1], [7]. An underlying assumption would seem to be that the burden of an episode of malaria disease is the same irrespective of when, or whether the episode was treated.

In most of sub-Saharan Africa, presumptive treatment (without prior diagnosis) has been the norm, and the number of treatments is a poor measure of the number of episodes (Table 1) and cannot be used to estimate disease burden. An increasing number of countries have adopted a policy of providing parasitological diagnosis, but this is not yet standard practice continent wide [1]. Instead of using problematic passive case detection data, maps of *P. falciparum* prevalence determined from surveys are combined with information on climate suitability for malaria transmission and population density in order to classify populations according to endemicity level. Clinical incidence values are assigned to each endemicity level based on estimates of the numbers of events recorded in longitudinal surveys of febrile malaria episodes in children, detected either actively or passively [8]–[10]. These estimates of populations at risk and endemicity-specific estimates of disease rates are together being used to produce national and continent-wide estimates of the number of clinical malaria episodes [11]. Longitudinal studies of malaria must always involve treating the acute episodes that are discovered, and subsequent to effective treatment, all the burden of disease potentially caused by the infection is averted. Treatment also reduces onward transmission to mosquitoes. In several longitudinal studies [12], [13], dramatic decreases in fever rates over time have been observed, presumably for these reasons. Intensive research studies are therefore likely to substantially underestimate clinical attack rates in the general population.

An alternative to these approaches is to use recalls of illness from cross-sectional surveys carried out in the community. An increasing body of data is available from demographic and health surveys (DHS), multiple indicator cluster surveys (MICS), and malaria indicator surveys (MIS), which include asking respondents to provide a recall of illness during the previous two weeks for each of their children. This paper shows how these data can be used to obtain improved estimates of the malaria disease burden.

## Methods

### Data Types

**Figure 2:**
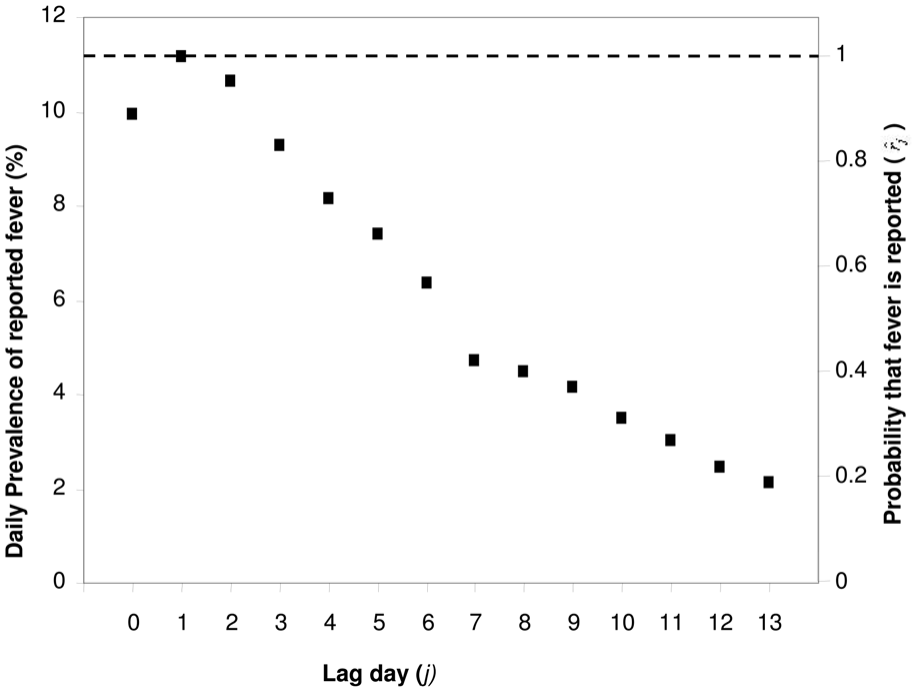
Recall of fever for each day in the 2 weeks prior to home visit, children aged <5 years, Asembo, Western Kenya. Left hand vertical axis: percentage of child population for whom fever was recalled; Right hand vertical axis: estimated recall probability. Source: [14].

Four types of data were used to illustrate the methods:

1. **Daily fever prevalence.** For the analysis of bias in recall of fever, data of Feikin and colleagues [14] for children under five years of age in Asembo, Bondo District, Kenya were used. These data comprise recalls of fever, elicited separately for each day in the reference period of fourteen day duration in a survey of approximately 25,000 people (Figure 2).
2. **Malaria therapy data.** Fever patterns in untreated and inadequately treated malaria patients were analyzed using the data of 330 neurosyphilis patients treated with *P. falciparum* in the National Institutes of Health laboratories in Columbia, South Carolina and Milledgeville, Georgia in the United States of America [3]. For each of these patients, the days on which fever (core temperature > = 39.4°C) occurred were recorded.
3. **Malaria Indicator Survey data.** Data on history of fever in the last fourteen days from the 2010 MIS from Zambia [15] were used. Just over 34% of children under five reported a fever in the last two weeks. This is period prevalence of fever, biased by recall. Of these, 34% took an antimalarial drug.
4. **Health Management Information System records.** Counts of diagnosed malaria patients from all health facilities in Luangwa District, Zambia extracted from the national HMIS database.

### Estimation of Recall Bias

Two-week morbidity recalls do not elicit complete information about illness during the reference period because respondents may forget or conceal information. The usual survey procedures do not directly provide any information about recall bias but it can be estimated when respondents are asked (individually) about illness on each distinct day during the reference period. This is because independence of the timing of the survey and the illness justifies the presumption that variations in fever rates by recall lag reflect recall bias.

The relative frequencies of fever reports by lag-day in the Asembo data provide a direct estimate of the recall bias associated with a specific lag in a recall (Figure 2). Assuming that a fever on the previous day is reported with 100% sensitivity, an estimate of the recall probability for a fever *i* days prior to interview is 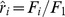 where *F_i_* is the fever prevalence recorded in the survey for the single day, *i* days prior to interview. (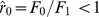 because surveys are usually carried out early in the day, before all fevers are yet evident.).

**Figure 3:**
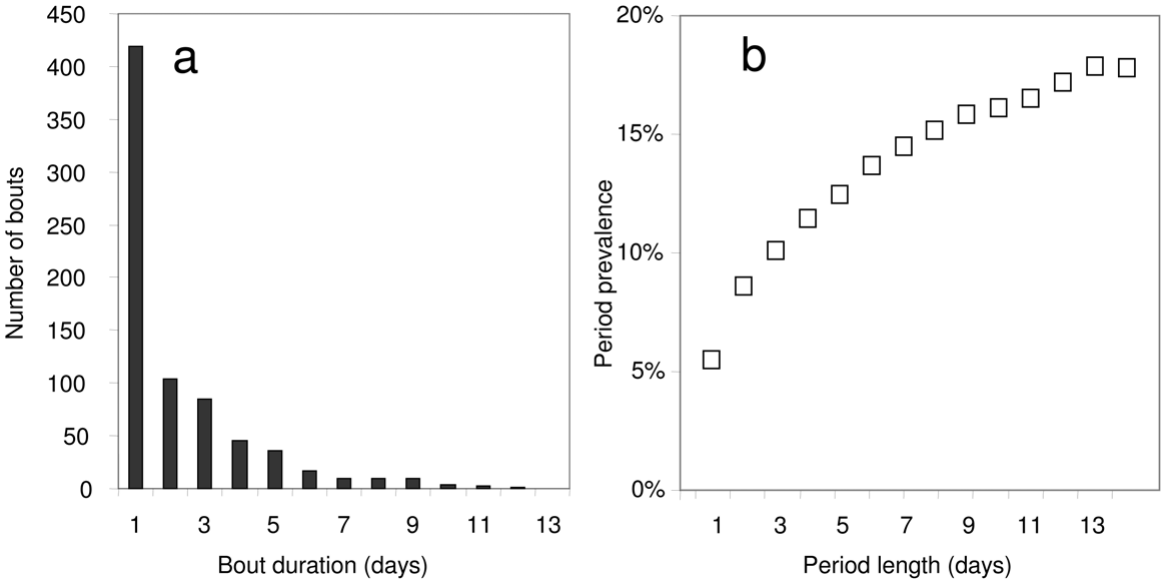
Effect of bout duration on period prevalence in the malaria therapy data. a: distribution of durations of uninterrupted bouts of fever in the malaria therapy data; b: period prevalence of malaria fever in the malaria therapy patients, as a function of the duration of the period.

Naively, the probability that a survey respondent reports fever, conditional on fever having occurred during a two week reference period might be thought to be 
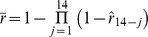
, however, fever bouts extend over multiple days (Figure 3a), and there may be multiple bouts during a single reference period (Figure 1), so the overall recall bias depends on the natural history of the disease. An estimate of the overall recall probability for a two week period, allowing for these effects, 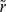, was obtained by applying the estimates of 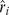 obtained from children in the Asembo study, to simulated interviews of malaria therapy patients, on the assumption that the number and pattern of days with fever during an arbitrary fourteen-day interval was similar in the field to those recorded in malaria therapy. The full recorded follow-up periods for malaria therapy patients were divided into fourteen-day intervals during which there was daily monitoring, leading to a total of 3715 fourteen-day intervals, during 755 of which there were one or more days with fever. Data were discarded for days that could not be included in these intervals because of gaps in, or termination of, the patients’ follow-up periods. For the analysis of recall in the absence of treatment, each day (*j* = 1, 2, …, 14) in each of these intervals was evaluated as though the patient had been interviewed at *j* = 14. Each day of fever was assumed recalled with probability 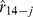 (obtained from children in the Asembo study) so that the probability that any fever was recalled in the simulation was 
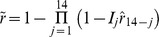
 where 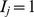 if there was fever on day *j* and 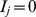 if there was no fever on day *j.*

### Estimation of Period Prevalence of Clinical Malaria from Survey Data

MIS use two-week reference periods to elicit histories of both illness and of treatment. The signs and symptoms of malaria are common to those of other diseases, so interviews alone perform poorly in assigning malaria as the cause of illness. To determine the proportion of recalled illness that is due to malaria, the results of parasitological testing are needed in addition to clinical data. In fourteen day recall surveys, individuals who report malaria fever are not necessarily parasitaemic at the time of the survey. However, rapid diagnostic tests (RDTs) based on the presence of the *P. falciparum* histidine rich protein 2 (*Pf*HRP2) measure the period prevalence of malaria infections. As *Pf*HRP2 persists in the bloodstream for up to a month following parasite clearance [16], [17], *Pf*HRP2 positivity (unlike blood slide positivity) can be used to detect recalled (as well as current) malaria fevers.

Some RDT-positive fevers are of non-malaria etiology, but have incidental parasitaemia. The proportion of fevers that are of malaria etiology (the malaria attributable fraction) can be estimated from the excess risk of fever among parasitaemic individuals, and must also be considered in the analysis.

Two approaches were used to obtain estimates of the period prevalence of malaria attributable fever, taking into account all the above considerations:

#### (i) Plug-in model for the period prevalence of malaria fevers

In this approach, the survey data from Luangwa, Zambia (Table 2) were used to separately estimate the malaria prevalence among fever recalls, *p_e_*, the period prevalence of reported fever, *p_f_*, and the relative risk of fever associated with malaria, *RR*, in each case without accounting for reporting bias, using formulae given in Table 3. The population attributable fraction of fever, *PAF*, and period prevalence of reported malaria attributable fever, *p_mf_*, were then obtained by substituting the estimates of *p_e_, p_f_*, and *RR* into further formulae given in Table 3. The point estimate of *p_mf_* thus obtained was then mapped on an estimate of the period prevalence of malaria fever allowing for reporting bias, 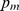, using further equations in the recall probability, for which the point estimate of 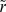 (described above) was substituted for *r* (formulae also in Table 3).

**Table 2:** Survey outcomes and their probabilities (children in Zambia).

RDT positive	Fever reported	TreatmentReported	Probability in branching process model (Figure 4)	Frequency in district survey	Frequency in national MIS*
No	No	–	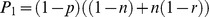	355 (62.6%)	
No	Yes	–	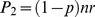	131 (23.1%)	
Yes	No	–	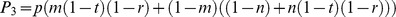	35 (6.2%)	
Yes*	Yes	No	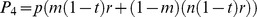	46 (8.1%)	686
Yes*	Yes	Yes	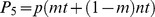		353

* The report of the national MIS does not distinguish antimalarial drug use depending on RDT result. Since only 16.7% of children with fever were reported to have received a parasitological diagnosis, we assume for the present analysis that antimalarial drug use was independent of RDT result.

**Table 3:** Plug-in model for period prevalence of malaria fever (equations and parameter estimates).

Symbol	Description	Source or equation	Estimate (95% credible interval)*
*r*	Probability that fever is recalled#	Asembo and malaria therapy data	0.81 (0.78–0.84)
*p_e_*	Prevalence of malaria among fever recalls	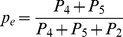	0.260
*RR*	Relative risk of fever associated with malaria	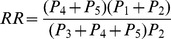	2.12
*PAF*	Proportion of all fever attributable to malaria (i.e. population attributable fraction of fever)	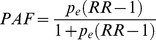	0.225
*p_f_*	Period prevalence of reported fever	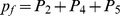	0.312
*p_mf_*	Period prevalence of reported malaria attributable fever	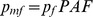	0.070
*r_f_*	Proportion of histories of fever reported	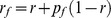	0.869
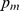	Period prevalence of malaria fever$	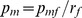	0.081

* Bayesian credible intervals computed assuming 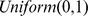 prior.

# allowing for reporting bias.

$ correcting for reporting bias.

This approach is easy to implement but does not allow for the different amounts of information available for the different outcomes. It does not readily provide interval estimates for the parameters.

#### (ii) Bayesian analysis of branching process model

**Figure 4:**
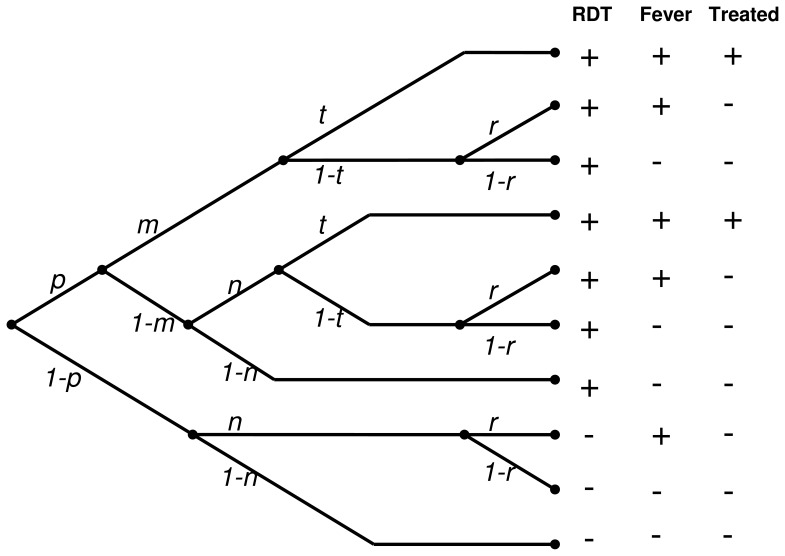
Branching process of events underlying cross-sectionally recorded outcomes. *p* is the probability of an RDT being positive; *m* is the probability of clinical malaria during any two week period, conditional on infection; *n* is the probability of non-malaria fever during any two week period; *t* is the probability of treatment with an antimalarial conditional on being both infected and febrile during the two-week period; and *r* is the probability that an untreated fever is reported.

The second approach was to analyse the determinants of both questionnaire and RDT outcomes as a branching process, as shown in Figure 4, where the columns ‘RDT’, ‘Fever’, and ‘Treated’ indicate the outcomes recorded at the survey, and the branches correspond to a classification of respondents according to whether they are *Pf*HRP2 positive, whether they suffered a malaria or non-malaria fever in the reference period, whether they received treatment, and whether the fever was reported at the survey.

Malaria fevers and non-malaria fevers are not distinguishable at the individual level in field data, so the ten branches shown in Figure 4 correspond to the five observable categories of outcomes, with probabilities 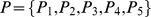 given in Table 2. The branching process analyses these probabilities as functions of the parameters *p*, *m*, *n*, and *t* (Table 2). A Markov chain Monte Carlo method (using WinBUGS v1.4 [18]) was used to obtain interval estimates for these parameters assuming 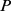 to follow a multinomial distribution. The WinBUGS code for fitting this model is included as supporting information (Script S1).

To complete the Bayesian model specification 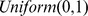 priors were used for *p*, *m*, *n*, and *t*. The distribution of 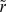 estimated from the malaria therapy data, was used as a prior distribution for *r*.

This makes all the parameters in the decision tree identifiable, conditional on the assumptions that the duration and frequency of bouts of fever in untreated individuals was the same in the two datasets; that treatment with anti-malarial drugs is relevant only in parasite positive individuals; that treatment never occurs in the absence of illness; and that respondents who fail to report illness are otherwise indistinguishable from those that report. A further assumption is that recall bias in treated cases is negligible, but that untreated fevers are recalled with some probability, *r*<1.

### Estimation of the Number of Days with Illness

**Figure 5:**
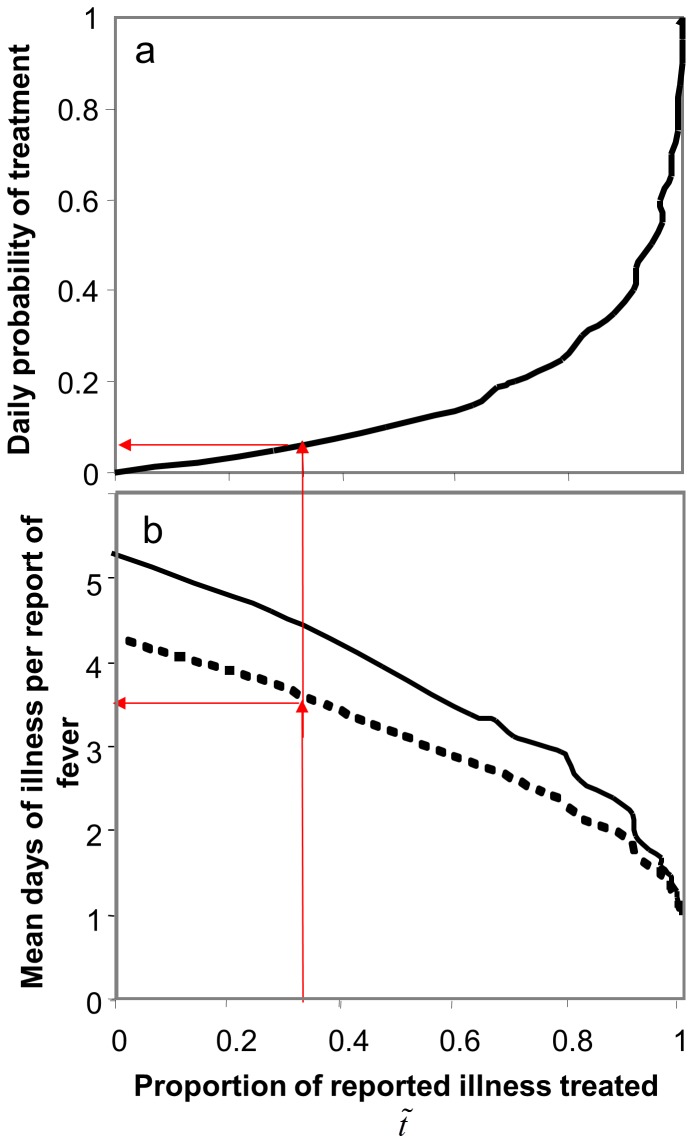
Analysis of impact of simulated treatment on morbidity data. The plots were constructed by simulating effective treatments applied to malaria therapy data (see Methods) with fourteen-day morbidity reference periods, a: daily probability that a patient receives treatment that day, given that s/he has a fever that day. The simulations take into account under-reporting, as estimated from the Asembo data, and assume an equal probability of commencing treatment on each day of illness. The arrow corresponds to the estimate made from the Zambian MIS data. b: mean days with illness per report of fever; continuous line: days with fever including fever that was not reported (adjusting for under-reporting estimated from the Asembo data); dashed line: days with fever during intervals for which fever was reported (ignoring recall bias).

Analyses of malaria therapy patients, with simulation of surveys and treatments (Figure 5), were used to estimate the numbers of days with illness associated with each recall of fever, conditional on the proportion of these (in the MIS) reporting treatment, 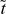. For a series of different daily probabilities of treatment, 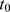, simulated treatments were assigned stochastically to each day of fever in the malaria therapy dataset. The simulated treatments were assumed to be completely effective, so that in the simulations, the patient had no more fever once treatment was initiated. (In contrast, the true treatments administered to the malaria therapy patients, predominantly at sub-therapeutic doses, were ignored in this analysis). Simulated surveys were implemented in which the data for fourteen-day periods were summarized assuming a level of recall bias determined from the Asembo data.

These simulations provided estimates of the numbers of days with illness per fever report, the overall level of underreporting, the proportion of recalls reporting treatment among those reporting illness, and the value of 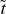, for each value of 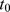. Each of these quantities could thus be plotted as a function of 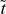 (Figure 5), and by reading off the values of each of them corresponding to the value of 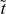 reported in the Zambian MIS, estimates could be made for the Zambian example, albeit under the assumption that the fever pattern in young children is similar to that of non-immune adult malaria therapy patients.

Since the MIS surveys were conducted only at one time of the year, the incidence estimates also needed to be adjusted for the effect of seasonal variation in clinical incidence. This was achieved by multiplying the estimate of the per capita number of days with malaria fever during the reference period by the ratio of the incidence of diagnostically confirmed cases in the HMIS during that period to the annual average incidence (Table 4).

**Table 4:** Parameter estimates for branching process model for period prevalence of malaria fever.

	Description	Estimates (95% credible interval)
*r*	Probability that an untreated fever is recalled#	0.81 (0.78–0.84)
*p*	Period prevalence of malaria infection	0.14 (0.12–0.17)
*n*	Period prevalence of non-malaria fever allowing for reporting bias	0.33 (0.29–0.39)
*t*	Proportion of periods with malaria fever that were treated	0.29 (0.27–0.32)
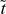	Proportion of malaria positive recalls where treatment was received	0.34 (0.31–0.37)
*m*	Probability of malaria fever conditional on infection	0.48 (0.29–0.67)
*p_m_*	Period prevalence of malaria fever allowing for reporting bias	0.069 (0.040–0.101)

# estimated from the Asembo and malaria therapy data.

## Results

### Recall Bias and Duration of Bouts

The daily prevalences of fever reported by Feikin and colleagues [14] clearly indicate that fevers which occurred a few days prior to survey are much less likely to be recalled than those which occurred the previous day, while fever is less likely to be reported on the day of interview (Figure 2). It is noteworthy that data from Papua New Guinea [19] show a similar pattern.

If each recalled bout of illness only entailed only day of fever, then a simple mean of the lag-day specific probabilities, 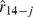, could be used to estimate the overall recall bias, which would be substantial. However, bouts of fever (as defined in Table 1) frequently last several days in malaria therapy patients (Figure 3a), and if these are not treated, there may be several bouts in one reference period, so that, while the proportion of days with fever is 5.4%, only 17.8% of two week periods include one or more days of fever (Figure 3b), instead of 54.0% if all bouts had lasted one day and were randomly spread over patients and follow-up periods.

Each additional day of fever adds to the probability that the bout will be recalled, so that when the lag-day specific recall probabilities for children from the Asembo study are applied to the patterns of fever occurrence in the malaria therapy data, 612 out of 755 (81%) fourteen day intervals with fever days are estimated to have been (hypothetically) recalled as containing one or more fever bouts, corresponding to 19% underreporting and a value of *r* = 0.81 (Table 4). This value of *r* is used in the estimation of 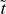 (see above).

The probability that fever is recalled is further complicated by the effects of treatment. While Figure 3 provides a description of the actual malaria therapy data, simulation of treatments under the assumption that a treated fever will always be recalled increases the probability that morbidity will be recalled in the simulations, while decreasing the corresponding number of days with fever (Figure 5).

The proportion of fever recalls mentioning treatment has a non-linear relationship with the daily probability of treatment, because recurrent fevers provide multiple opportunities to treat, so even a modest rate of treatment per day of fever will result in a very high proportion of recalled fevers being treated. The simulation of surveys based on the malaria therapy data suggests that the 34% of fever recalls in the Zambian MIS data that reported treatment (Table 2) correspond to only about 6.6% treatment per day of fever (Figure 5a). The proportion of treatments delivered promptly is not the same as the daily probability of treatment because prompt treatment (as defined in the MIS questionnaire) may occur on either the same day, or the day after onset of fever. A 6.6% daily probability that a fever will be treated consequently corresponds to a probability almost twice as high as this that treatment will occur in the first two days, which is comparable with, though somewhat lower than, the 18.7% of fever reports that indicated receipt of prompt anti-malarial treatment in the survey, (*t_e_* = 0.187).

### Period Prevalence of Clinical Malaria

Table 2 gives the number of respondents in the Luangwa district malariological survey in each of the four classes categorized by RDT result and fever report. The observed proportions in each category were used to obtain estimates of the period prevalence of malaria fever firstly by calculating each of the quantities given in Table 3, substituting the data from Table 2 (the plug-in approach). This provided a period prevalence estimate of 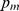 = 8.1% (Table 3).

A second period prevalence estimate of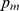 = 6.9% (95% credible interval 4.0% –10.1%) (Table 4) was obtained using the Bayesian analysis of the branching process, which also provided interval estimates of *p*, *m*, and *n.* All these estimates were conditional on the distribution of *r* estimated from the Asembo data on recall probabilities, and on the analysis of total fever days in the malaria therapy data (Figure 3). The analysis of the branching process implied that of the 14% of children with evidence of malaria parasites, about half of them had suffered malaria fevers during the two-week reference period. Of those who mentioned fevers in the preceding two weeks, treatment was mentioned by 34%. However, only 29% of children with fever were estimated to have been treated at some point in the period, because of bias in the recall of untreated fevers.

### Number of Days with Fever Associated with each Fever Positive Reference Period

The number of days with fever associated with each reference period reported to have fever, depends on the treatment rate. As treatment rates increase, the number of days with fever corresponding to each report decreases, since an increasing proportion is averted by the treatments, until in the limiting case of 100% prompt and effective treatment, each report corresponds to exactly one day with fever (Figure 5b). In the absence of treatment, the 755 two-week periods of malaria therapy with at least one day of fever had a mean of 4.3 days with fever each.

The recalled treatment rate for a positive RDT together with a fever estimated from the national level data is 34.0% (right hand column of Table 2). The simulations of treatment using the malaria therapy data imply that this treatment rate corresponds to a mean of 3.6 days with recalled illness in each interval for which fever was recalled (from the results shown as dashed line in Figure 5b). However, based on the analysis above, 19% underreporting of untreated fevers is assumed, implying that there are 4.4 days of fever in the population for every positive fourteen day reference period (read from continuous line in Figure 5b).

### Total Burden of Uncomplicated Malaria for Zambian Children

**Figure 6:**
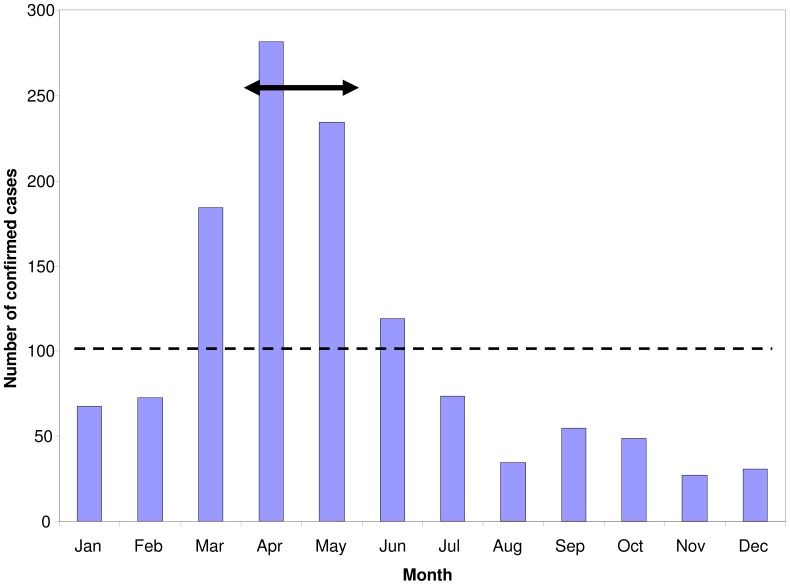
Average number of confirmed cases by month in Luangwa District Zambia 2009–2010. The dashed line corresponds to the annual average number of cases per month and the double headed arrow to the survey period and the corresponding average incidence.

Figure 6 shows the seasonality in confirmed malaria cases at all health facilities in Luangwa District, Zambia, from HMIS records. The MIS surveys in Luangwa were conducted during peak transmission season (April-May) and thus the annual burden estimate needs to be scaled by the ratio of RDT confirmed malaria fever incidence over the whole year, relative to the incidence during this period (Table 5). It is assumed that this district is representative in terms of the degree of seasonality, and the targeting of the MIS surveys to the peak malaria season. If this district is representative, this provides an estimate of the average number of days with malaria fever per person-year in Zambian children of 2.6 days (95% credible interval: 1.5–3.8) of malarial fever per person at risk per year (Table 5).

**Table 5:** Conversion of estimated period prevalence of malaria fever to estimate of disease burden.

Symbol	Description	Source or equation	Estimate
*t_0_*	Daily probability of treatment	Read from Figure 5a as a function of 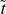	0.066
*t_e_*	Probability of prompt and effective treatment	MIS data	0.187
*d*	Days with malaria fever during reference period among those who report malaria fever	Read from Figure 5b as a function of 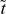	3.58
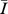	Annual average incidence of confirmed clinical malaria at health facility (cases per month)	HMIS data from Luangwa District (Figure 6)	102.3
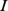	Incidence of confirmed clinical malaria at health facility (cases per month) during period of MIS survey	HMIS data from Luangwa District (Figure 6)	258.0
*b*	Days with malaria fever per person-year at risk	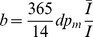	3.0#2.6* (1.5–3.7)

# Based on the plug-in model estimate of *p_m_*.

* Based on the branching-process model estimate of *p_m_*. Figures in parentheses give the 95% credible interval obtained by treating *d,*_
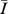
and 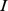
as fixed quantities._

This compares with an incidence estimate of 1.5 million cases in the year 2009 (confirmed and probable) for the 2.3 million children under five years old in Zambia given in the World Malaria Report [20], corresponding to 0.65 cases per capita. Using the estimate of 2.6 days with fever per capita, this implies, each recorded case corresponds to about 4 days with fever in the community. This all seems plausible, though the credible intervals (which capture most of the uncertainties in the data) do not capture the full level of uncertainty implied by the assumptions that patterns of fever in Zambian children may be similar to those in malaria therapy patients, that recall patterns in Kenya can be applied to Zambia, and that clinical malaria attacks will always lead to a positive RDT if the test is applied within fourteen days of a fever bout.

## Discussion

Quantification of malaria in an area can have various objectives. This paper focuses on the assessment of disease burden, defined in terms of experience of illness. This is needed for assessing progress in improving health and for cost-effectiveness analyses of intervention programs. Disease burden quantification requires population-based data, and community-based measures of parasite prevalence and anaemia prevalence, available from MIS, are recommended as morbidity indicators for national malaria control programs in the monitoring and evaluation (M&E) toolkit of the Global Fund to fight AIDS, TB and Malaria (GFATM) [21]. However, parasite prevalence and anaemia prevalence are not direct measures of malaria morbidity. They are multifactorial [22] and may change at different rates than clinical malaria incidence does [23].

In this paper, fever recalls from MIS surveys are proposed as the primary source for estimates of malaria morbidity burden. This approach contrasts with the use of HMIS data as the primary source for estimates of disease burden. From the clinical perspective, it may be good enough to identify an “episode” or “case” when an individual presents to a health facility with febrile illness and detectable parasitaemia. Activity statistics derived directly from such case registration are useful for commodity forecasting and managing clinical workloads, and may also be suitable as outcomes in intervention trials where the goal is simply to detect a difference between two or more arms, but these are only a subset of the reasons for quantifying the amount of malaria, and in particular are not the same as assessment of disease burden. Other possible objectives include assessment of the level of transmission (which requires consideration of asymptomatic infections as well as clinical attacks [24]), and measurement of the economic burden of the disease (which needs to consider also the costs of diagnosing malaria negative patients, of preventive measures, and indirect costs of illness, including productivity costs and loss of investment because of concerns about the disease).

If activity statistics (such as HMIS) data are used as the primary source of data on disease burden, they need adjustments. Firstly, the calculations must correct for sick individuals who do not report to the health facility. Making this adjustment is challenging, because untreated malaria attributable fevers that are invisible in the statistics have different durations from the treated fevers that are visible in the statistics. Adjusting for this is especially challenging when there are changes in access to care, since improved access reduces disease burden non-linearly while increasing levels of activity. Additionally, it is not clear how to adjust activity statistics to allow for the benefits of improvements in case management that replace failing treatments and reduce the duration of illness, such as the roll-out of ACT as first-line therapy across Africa. Lastly, activity statistics do not distinguish changes in diagnostic accuracy from changes in epidemiology.

Thus, until recently in places highly endemic for malaria, a febrile patient with a viral infection was generally presumptively treated for malaria and recorded as a malaria case. Control of malaria and the introduction of parasitological diagnoses mean that such patients are now less likely to appear in malaria statistics. Rather, they are more likely to be classed as respiratory infections. This is appropriate, insofar as these statistics measure the activity of the health system, but the introduction of diagnostic tests for malaria has not resulted in a larger burden of disease due to respiratory infections. Similarly, the proportion of non-malaria fever patients with incidental parasitaemia declines when malaria transmission is reduced. These patients should be treated for malaria, and hence contribute to activity statistics, but the burden of disease associated with them should be assigned to the etiological agent causing the fever, in such cases not malaria, so that changes in malaria burden estimate should be unaffected by the number of such patients. In general therefore, the use of activity statistics to measure burden can give a quite false idea of the importance of malaria relative to other illnesses and may misrepresent the relative contributions of preventive and curative interventions to improvements in public health.

Fever recalls from surveys provide a direct measure of morbidity, are increasingly widely available, and in some situations data have been validated as comparative morbidity measures [25]. This paper provides algorithms for converting such data into annualized malaria-specific disease burden (in days with illness). These calculations, however, need auxiliary information: some of this is also gathered as part of standard MIS protocols, specifically recalls of treatment and parasite prevalence assessed by RDT. HMIS data, which are needed for scaling the incidence estimates to adjust for the surveys’ timing in the year, are also widely collected though often only made available as aggregated annual statistics. The conversion of fever recalls to days with illness requires specific data on length and spacing of illness bouts only available from malaria therapy data, and on recall bias from a Kenyan field study. The overall approach can be applied to any site and time-period for which population-based surveys of fever recall and parasitaemia (by RDT) are available. In the application to Zambian data, the plug-in and branching process approaches for analysing the survey data provided similar but not identical estimates of the period prevalence of clinical malaria. The plug-in approach is probably easier to understand and implement, though the Bayesian approach makes more efficient use of the data. The plug-in approach could easily be adopted as a standard, since this would involve simply substituting local data for those from Zambia into the calculations in Tables 3 and 5.

The validity of combining these datasets can be questioned, especially because of the assumption that the patterns of fever in African children parallel those in malaria therapy patients. Superficially, patterns observed in closely monitored Gambian children [26] were similar to those in malaria therapy but the available field datasets are too small for any quantitative comparison. The assumption that RDTs provide good estimates of period prevalence of infection could also be questioned. However, the approach does lead to parameter estimates with at least face validity (Table 3), and unfortunately these are the only kinds of data available that provide all this information, so validation against definitive measures of burden is currently not possible. Despite these shortcomings, estimates of burden, based on this approach, would very likely be a substantial improvement on current practice in cost-effectiveness analysis, because they change in a qualitatively appropriate way in response to either preventive or curative interventions. This also has implications for simulation modeling of case management, which are discussed in the supporting information (Text S1). A next step should be to carry out this estimation for many more settings and to compare trends and geographical patterns with those based on current practice.

Some of the data limitations could be addressed by improvements in survey design. First, cross-sectional surveys of malaria illness and treatment-seeking need to include questions on history of fever and measure parasitaemia in all age groups, not just in children under five: as malaria control efforts are scaled-up and transmission falls, malaria illness tends to shift into older age groups [27] due to greater time to first exposure and thus slower acquisition of immunity. Burden is thus expected to be heavier in older children and adults, and monitoring systems need to allow for this reality in order to correctly capture the burden of malaria illness, both in absolute terms and in terms of change over time. Second, there is a need for more data like those from Asembo to estimate recall bias. Ideally, 24-hour recalls would be used, but this would reduce the size of available databases. Finally, the current practice of carrying out MIS at approximately the same time across whole countries means that there are limited data available on seasonality in either parasitological or clinical indices. Data for each period of the year are essential for unbiased estimates of annual burden, and could in principle be obtained by carrying out rolling surveys, visiting clusters in a random order throughout the year.

### Conclusion

Measurement of malaria burden is fraught with complexity mainly due to the natural history of the disease and to sub-optimal health facility utilization which means that treatment is often delayed or not sought. Definitions of malaria episodes are either ambiguous or difficult to use because good information about patterns of infection, recurrence of fever or asymptomatic infection is rarely available.

This paper suggests that the point prevalence of malaria attributable disease, or equivalently, the days with malaria fever in unit time, should be used as a measure of burden. This avoids the problem of defining a malaria episode, and suggests that burden can, in principle, be estimated in an unbiased way from data that are already collected in national MIS, combined with data on seasonality. The estimates used in this paper of recall bias, and of the numbers of days with illness contributing to single illness recalls, could be applied more generally.

It is hoped that this work will stimulate a dialogue on how to improve measurement of the burden of uncomplicated malaria.

## Supporting Information

Figure S1 **Mean duration of illness per episode depending on treatment in malaria therapy patients.** The mean number of fever days per episode (duration of illness) depending on the probability of treatment of fever and length of the health system memory for treated episodes (**a** & **d**) and untreated episodes (**b** & **e**), and the probability of an episode being treated (**c** & **f**), for daily probability of treatment (**a**–**c**), and five-daily probability of treatment (**d**–**f**). Health system memory length (days): black 5, red 10, green 15, dark blue 20, light blue 25, magenta 30, yellow 35, grey 40. Smoothing splines were drawn through scattered points, which were averages of 100 repeated simulations.
(TIF)

Figure S2 **Probability in micro simulation models of an episode being treated.** The probability of an episode being treated is plotted against the treatment seeking probability per five-day time step, with a 15 day health system memory. The green line is copied from Figure S1f and represents the results from the malaria therapy analysis on a non-immune population of adults. Each black line represents the median probability of an episode being treated for children under five years of age, with a health system memory of 15 days, out of 10 simulation runs for a given model variant. Each model variant represents a different set of assumptions about malaria transmission and epidemiology in terms of decay of immunity and heterogeneities in exposure, co-morbidity and/or access to treatment, [28], [29]. Simulations were done of a setting where the entomological inoculation rate was 20 infectious bites per adult per annum, with a total human population size of 10,000. Each red line represents the median probability of an episode being treated for the entire population for all 12 model variants. Two model variants from the original ensemble [30], *R0674* (uncorrelated heterogeneities in access to treatment and susceptibility to co-morbidity) and *R0678* (heterogeneity in access to treatment), are excluded because at high treatment coverage levels, there is an upper limit to the level of heterogeneity.
(TIF)

Text S1 **Simulation modeling of the burden of uncomplicated malaria.**
(DOC)

Script S1 **WinBUGS code for estimating parameters of branching process.**
(DOC)
