# Supplementary material for: A Novel Approach for Measuring the Burden of Uncomplicated Plasmodium falciparum Malaria: Application to Data from Zambia
Source: PLoS One. 2013 Feb 28;8(2):e57297. doi: 10.1371/journal.pone.0057297 (PMC3585385; doi:10.1371/journal.pone.0057297)
Supplement: Text S1 — Simulation modeling of the burden of uncomplicated malaria. (DOC) [file pone.0057297.s003.doc]

Simulation modeling of the burden of uncomplicated malaria

Studies that aim to predict long-term impacts and cost-effectiveness of novel intervention strategies are often best carried out by simulation modeling. Measures of disease burden in such studies need to be aligned with the data that can be obtained from field studies, and should use the same terminology and definitions, which disciplines the practitioner into using explicit definitions. Individual-based stochastic simulation models of the biology and epidemiology of *P. falciparum* malaria have been developed and applied to estimate the cost-effectiveness of scaling up case management [1,2]. These models went beyond previous static models (such as those used by the Global Forum for Health Research [3]), by including the effect of treatment in truncating an infection, and the dynamic effects of treatment on transmission.

For prediction of the health effects of scaling up of effective case-management, Tediosi and colleagues [2] define a quantity termed the ‘health system memory’, generally taking a value of 30 days, and consider all clinical events occurring within an interval of this length as part of the same episode. To compute disability-adjusted life years (DALYs) they then use a value for duration of illness of 0.01 years (3.65 days), and disability weights for episodes taken from the Global Burden of Disease (GBD) study [4], without critically evaluating the GBD meaning of a malaria episode. The same value for duration has been used in studies of intermittent preventive treatment [5], while a duration of 7 days has been used elsewhere [6]. It is unclear whether these episode weights are valid for the duration of illness (combined number of days with fever during an episode), or for the entire episode, which could last much longer and includes intermittent illness-free days. In the latter case, DALY calculation where the duration is for fever days (and not episode days) should probably use higher disability weights. More important in the analysis of the effects of scaling up case management, using a fixed duration of illness ignores the dependence of duration of illness (within the episode) on if and when treatment is administered. For the duration of illness in treated episodes, Snow and colleagues [7] use 5.1 days for patients in the 0–14 year age range and immunologically naïve people, while using 2.0 days for adults in stable endemic areas. Mueller and colleagues [8] use a value of two weeks for the duration of episodes, there possibly synonymous with duration of illness, without quoting empirical evidence.

Malaria therapy data can be used to estimate the number of days per episode on which fever occurs (duration of illness) for non-immunes, depending on the length of the health system memory and the treatment probability on each day of fever. Similar to the analyses described in the main text, but without including recall effects, analysis were carried out on the data from all those *P. falciparum* malaria therapy patients [9], for whom days with fever were recorded during the course of the infection. In these analyses, simulated treatments were applied to the observed sequences of fever and parasitaemia, with patients having a probability of being cured of malaria by simulated treatment on each day of fever. The ‘daily treatment probability’ was assumed to be independent of the fever history. Episodes were started on a day with fever and ended after the length of the health system memory, or earlier, on the day of treatment, if treatment occurred. The health system memory was varied between five and 40 days, with increments of five days, and the treatment probability on each day of fever was varied between zero and one, with increments of 0.025. The mean number of days with fever per episode, conditional on treatment, and the total number of episodes, also conditional on treatment, were recorded. If treatment occurred, the day of treatment was counted as a fever day, and the patient was cured. If treatment did not occur, the first fever day after the end of the previous episode started a new episode. This procedure was repeated 100 times, and the results were averaged.

Figure S1a shows the mean number of fever days per episode in treated individuals, depending on the health system memory and the daily treatment probability. With a daily treatment probability of zero, no episodes were treated. With increasing daily treatment probability, the number of days with fever that were experienced during an episode until treatment occurred was reduced. With a daily treatment probability of one, all episodes lasted only one day.

Similarly, Figure S1b shows the mean number of fever days per episode in untreated individuals, depending on the health system memory and the daily treatment probability. With a daily treatment probability of one, no episodes were untreated. With increasing probability of treatment, the mean number of fever days per untreated episode also fell, because the episodes with more fever days were more likely to be treated, and thus excluded from the category of untreated episodes.

Figure S1c shows the probability of an episode being treated, depending on the treatment probability on each day of fever and the length of health system memory. This probability was calculated as the number of treated episodes divided by the total number of episodes. The relationship between the treatment probability per episode (which is also the treatment coverage in a population) and the daily treatment probability of a fever is non-linear: the probability of an episode not being treated is equal to the daily probability of a fever not being treated (equal to one minus the daily treatment probability) raised to the power of the number of fever days in the episode.

The model employed by Tediosi and colleagues [2] is a discrete time representation of the dynamics of malaria in individuals using five-day time steps, implemented on the OpenMalaria platform (http://code.google.com/p/openmalaria/). Bouts of illness (see Table 1) are represented by a classifying each five-day time step according to whether it included any days with malaria fever. In publications based on these models [10–12], numbers of episodes are calculated by grouping together bouts occurring within 30 days of the first time step with fever. The grouping into one month periods follows an approach used in some field studies [13]. This period can be justified by reference to prophylactic periods associated with treatment and the duration of standard *in vivo* tests for drug resistance. It can be thought of as the period over which the patient or health care system considers the bouts of illness to be part of the same illness (Table 1).

In order to calculate the mean number of fever days during an uncomplicated episode in a five-day time step model using malaria therapy data, the daily treatment probability needed to be mapped onto a five-daily treatment probability. This could have been done by mapping the mean fever day-values in Figures S1a and S1b onto the probability of treating an episode for a five-day health system memory in Figure S1c, instead of onto the corresponding daily treatment probability. However, the analyses of malaria therapy data first needed to be adapted to a five-day time step system by grouping the daily data into five-day periods. In malaria therapy, days with fever were not homogenously spread over time, but tend to be more frequent near the beginning of the period of patent parasitaemia (Figure 1). This has an effect on the relationship between one-day and 5-day treatment seeking probabilities. If fever starts towards the end of a five-day period, there will be fewer fever days in the time step, and a daily treatment probability is associated with a slightly lower episode treatment probability. This effect was corrected for by randomly offsetting the starting day of the analysis.

Figures S1d and S1e show how the mean number of fevers days during malaria episodes depends on the five-daily treatment probability, for treated and untreated episodes, respectively.

The ‘reference’ health system employed by Tediosi and colleagues [2] uses a five-daily treatment probability of 5%, and a 30 day health system memory. With this treatment probability, the mean illness duration for a treated episode was 5.2 days. This is very close to the value of 5.1 days used by Snow and colleagues [7]. For an untreated episode, the mean number of fever days was 6.3 at these health system settings. This is a much shorter illness duration than the two weeks assumed in some other studies.

A five-daily treatment probability of 5% (corresponding to a 2.15% daily treatment probability), may seem very low. However, the incidence rates in these models were fitted to incidence data from the villages of Ndiop and Dielmo in Senegal [14,15], which were measured through daily surveillance carried out with the explicit intention of detecting (and treating) every clinical malaria attack. These rates were higher than reported in other studies. The number of treatments in MIS, MICS, or DHS is generally low compared to this high number of fever incident days, and low daily treatment probabilities may thus be justified. In the main text of this paper, a daily treatment probability of about 7% was estimated for Zambian children.

Figure S1 describes the duration of illness depending on treatment for a non-immune population of malaria therapy patients. The relationship between the mean number of fever days during episodes and the daily treatment probability will depend on how many fever days each new infection is expected to generate (in the absence of treatment). This depends heavily on immunity. In populations living in malaria endemic areas and with resulting acquired immunity, with a given probability of treatment per fever day, the probability of treatment per episode is lower because there are fewer fever days per episode. This is illustrated in Figure S2, which displays both the curve for a 15 day health system memory from Figure S1f and the results of a simulation study in a setting with an annual entomological inoculation rate (EIR) of 20 infectious bites per person per year. Whereas the curves for children under five years of age (who have little built-up acquired immunity) compares well to the curve of malaria therapy patients, especially for the part of the curve below a five-daily probability of 40%, the curves for the entire population are much lower. Not only does immunity affect the number of fever days per episode, it also alters the severity of fever, and hence the likelihood of treatment. As a result of this, within the same all-age population, more immune adults may have a lower treatment seeking probability than children.

HMIS, MICS and MIS routinely collect information on the percentage who took antimalarial drugs (PAMD) following the onset of fever, among children under five years of age with fever, as recalled by their care giver for the two weeks preceding the interview. These survey data can be converted into model time-step specific treatment probabilities for input into these models. Assuming that these survey data suffer from recall bias similar to that found in the Asembo data, Figure 5A in the main text can be used to convert the PAMD into the daily probability that if a fever occurs (on that day), the child is treated with anti-malarial drugs (on that day). Important assumptions are that the pattern of fevers in children is similar to that in the non-immune adults who received malaria therapy, and that the probability of treatment of a fever on a given day is independent of the fever history.

For five-day time step models that require parameterization of five-daily treatment probabilities, such as also implemented in OpenMalaria, the daily probability of treatment of a fever given that it occurs, can be converted into the five-daily probability that treatment occurs given that a fever occurs on one or more days during the five day period. This is done by mapping the daily treatment probability onto the black line in Figure S1c. On the OpenMalaria website [http://code.google.com/p/openmalaria/], an example R script can be downloaded for converting between HMIS type data and model parameter values.

For five-day time step models, the fourteen day reference period was approximated by setting the health system memory to 15 days. Figure S2 illustrates that similar to the relationship between daily and five-daily treatment probabilities, the relationships between fourteen day treatment probabilities and daily or five-daily treatment probabilities are non linear. The relationship varies slightly depending on the model variant.

## Reference List

1. Smith T, Maire N, Ross A, Penny M, Chitnis N, Schapira A, Studer A, Genton B, Lengeler C, Tediosi F, de Savigny D, Tanner M (2008) Towards a comprehensive simulation model of malaria epidemiology and control. Parasitology 135: 1507-1516. 10.1017/S0031182008000371.

2. Tediosi F, Maire N, Smith T, Hutton G, Utzinger J, Ross A, Tanner M (2006) An approach to model the costs and effects of case management of Plasmodium falciparum malaria in sub-Saharan Africa. Am J Trop Med Hyg 75: 90-103.

3. Goodman, C. A., Coleman, P. G., and Mills, A. (2000) Economic analysis of malaria control in sub-Saharan Africa. Geneva: Global Forum for Health Research.

4. Murray, C. J. L. and Lopez, A. D. (1996) The global burden of disease: a comprehensive assessment of mortality and disability from diseases, injuries, and risk factors in 1990 and projected to 2020. Harvard: Harvard University Press.

5. Sicuri E, Bardaji A, Nhampossa T, Maixenchs M, Nhacolo A, Nhalungo D, Alonso PL, Menendez C (2010) Cost-effectiveness of intermittent preventive treatment of malaria in pregnancy in southern Mozambique. PLoS ONE 5: e13407. 10.1371/journal.pone.0013407 [doi].

6. Kumar A, Valecha N, Jain T, Dash AP (2007) Burden of malaria in India: retrospective and prospective view. Am J Trop Med Hyg 77: 69-78. 77/6_Suppl/69 [pii].

7. Snow R, Craig M, Newton C, Steketee RW (2003) The public health burden of *Plasmodium falciparum* malaria in Africa: Deriving the numbers.

8. Mueller DH, Wiseman V, Bakusa D, Morgah K, Dare A, Tchamdja P (2008) Cost-effectiveness analysis of insecticide-treated net distribution as part of the Togo Integrated Child Health Campaign. Malar J 7:73.: 73.

9. Collins WE, Jeffery GM (1999) A retrospective examination of the patterns of recrudescence in patients infected with *Plasmodium falciparum*. Am J Trop Med Hyg 61: 44-48.

10. Maire N, Tediosi F, Ross A, Smith T (2006) Predictions of the epidemiologic impact of introducing a pre-erythrocytic vaccine into the expanded program on immunization in sub-Saharan Africa. Am J Trop Med Hyg 75: 111-118.

11. Tediosi F, Maire N, Penny M, Studer A, Smith TA (2009) Simulation of the cost-effectiveness of malaria vaccines. Malar J 8: 127.

12. Penny MA, Maire N, Studer A, Schapira A, Smith TA (2008) What should vaccine developers ask? Simulation of the effectiveness of malaria vaccines. PLoS ONE 3: e3193.

13. Alonso PL, Smith TA, Armstrong-Schellenberg JR, Kitua A, Masanja H, Hayes R, Hurt N, Font F, Menendez C, Kilama WL, Tanner M (1996) Duration of protection and age-dependence of the effects of the SPf66 malaria vaccine in African children exposed to intense transmission of Plasmodium falciparum. J Infect Dis 174: 367-372.

14. Rogier C, Trape JF (1995) [Study of premunition development in holo- and meso-endemic malaria areas in Dielmo and Ndiop (Senegal): preliminary results, 1990-1994[]. Med Trop (Mars) 55: 71-76.

15. Smith T, Ross A, Maire N, Rogier C, Trape JF, Molineaux L (2006) An epidemiologic model of the incidence of acute illness in Plasmodium falciparum malaria. Am J Trop Med Hyg 75: 56-62.

16. Feikin DR, Audi A, Olack B, Bigogo GM, Polyak C, Burke H, Williamson J, Breiman RF (2010) Evaluation of the optimal recall period for disease symptoms in home-based morbidity surveillance in rural and urban Kenya. Int J Epidemiol 39: 450-458.

17. Maire N, Tediosi F, Ross A, Smith T (2006) Predictions of the epidemiologic impact of introducing a pre-erythrocytic vaccine into the expanded program on immunization in sub-Saharan Africa. Am J Trop Med Hyg 75: 111-118.

18. Smith T, Ross.A., Maire N, Chitnis N, Studer A, Hardy D, Brooks A, Penny M, Tanner M (2012) Ensemble modeling of the likely public health impact of a pre-erythrocytic malaria vaccine. PLoS Med 9: e1001157.
