# Supplementary material for: A Novel Approach for Measuring the Burden of Uncomplicated Plasmodium falciparum Malaria: Application to Data from Zambia
Source: PLoS One. 2013 Feb 28;8(2):e57297. doi: 10.1371/journal.pone.0057297 (PMC3585385; doi:10.1371/journal.pone.0057297)
Supplement: Script S1 — WinBUGS code for estimating parameters of branching process. (DOC) [file pone.0057297.s004.doc]

# WinBUGS code for estimating parameters of branching process.

Model

{

# For the treatment probability use the national MIS dataset

Prt[2]<- p*(m*(1-t)*r+(1-m)*(n*(1-t)*r))

Prt[1]<- p*(m*t+(1-m)*n*t)

ttilde <- Prt[1]/(Prt[1]+Prt[2])

nt~dbin(ttilde,Nt)

# For the relationship of RDTs and morbidity use the district dataset.

Pr[1]<- (1-p)*((1-n)+n*(1-r))

Pr[2]<- (1-p)*n*r

Pr[3]<- p*(m*(1-t)*(1-r)+(1-m)*((1-n)+n*(1-t)*(1-r)))

Pr[4]<- Prt[1]+Prt[2]

N_dis<- sum(n_RDT[])

n_RDT[1:4]~ dmulti(Pr[1:4],N_dis)

total<- Pr[1]+Pr[2]+Pr[3]+Pr[4]

# For the recall probability use the analysis of the Asembo and malaria therapy data.

nr ~dbin(r,Nr)

# To calculate burden multiply:

d<- 3.66

pm<- p*m

b<- 365/14*d*p*m*Ibar/I

# Uniform priors for probabilities

p~ dunif(0,1)

n~ dunif(0,1)

m~ dunif(0,1)

r~ dunif(0,1)

t~ dunif(0,1)

}

Data

list(n_RDT=c(355,131,35,46), nr=612, Nr=755, nt=353, Nt=1039, Ibar=102.3, I=258.0)
